# Supplementary material for: Colonization of methicillin-resistant Staphylococcus aureus and vancomycin-resistant Enterococci and its associated factors in cancer patients at the University of Gondar Comprehensive Specialized Hospital, Northwest Ethiopia
Source: PLoS One. 2025 Feb 7;20(2):e0318242. doi: 10.1371/journal.pone.0318242 (PMC12140114; doi:10.1371/journal.pone.0318242)
Supplement: S1 Fig — Legend: MRSA, methicillin resistant Staphylococcus aureus; MIC: minimum inhibition concentration; VISA: vancomycin intermediate Staphylococcus aureus. (PDF) [file pone.0318242.s001.pdf]

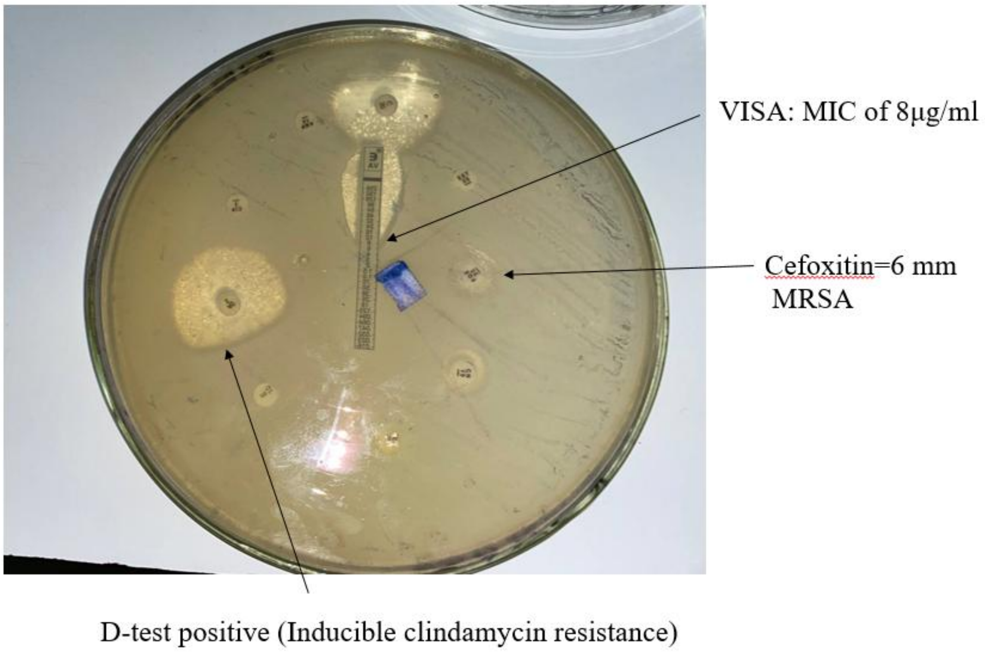

S1 Fig. D-test positive (Inducible clindamycin resistance) methicillin resistant *Staphylococcus aureus*. Legend: MRSA, Methicillin resistant *Staphylococcus aureus*; MIC: minimum inhibition concentration; VISA: vancomycin intermediate *Staphylococcus aureus*.
