# Supplementary figures and images for: Colonization of methicillin-resistant Staphylococcus aureus and vancomycin-resistant Enterococci and its associated factors in cancer patients at the University of Gondar Comprehensive Specialized Hospital, Northwest Ethiopia
Source: PLoS One. 2025 Feb 7;20(2):e0318242. doi: 10.1371/journal.pone.0318242 (PMC12140114; doi:10.1371/journal.pone.0318242)

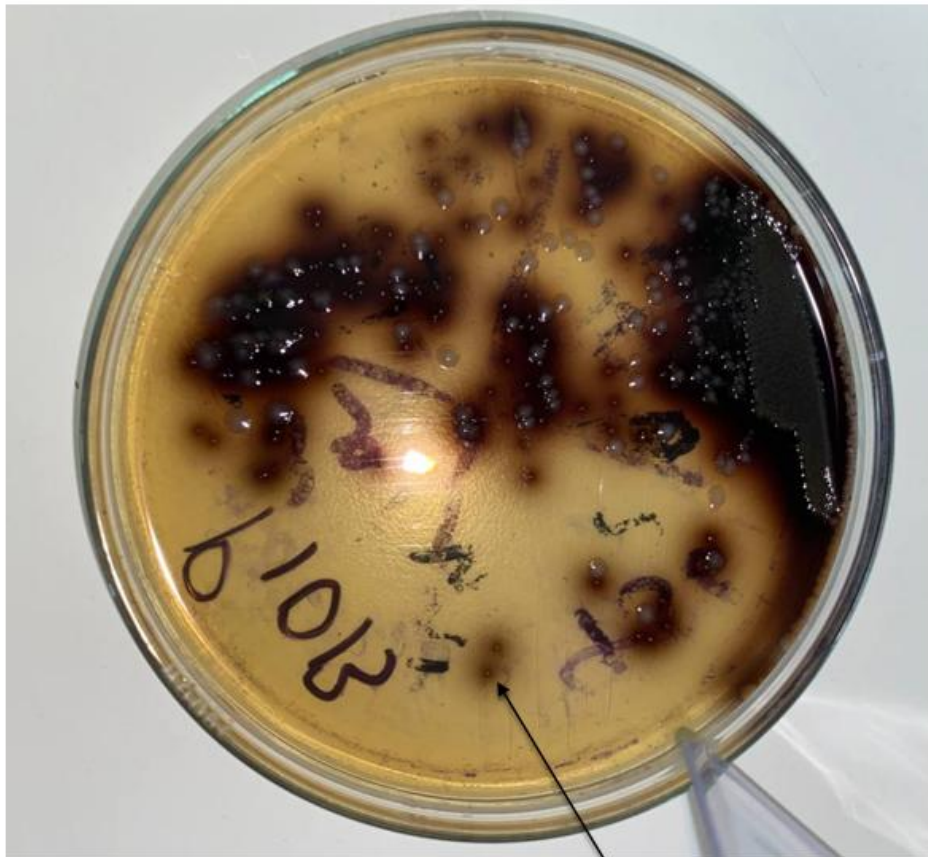

*Enterococcus* species on BEA

S2 Fig. *Enterococci* colony on Bile esculin agar

Supplement: S2 Fig — (PDF) [file pone.0318242.s002.pdf]
