# Supplementary material for: Colonization of methicillin-resistant Staphylococcus aureus and vancomycin-resistant Enterococci and its associated factors in cancer patients at the University of Gondar Comprehensive Specialized Hospital, Northwest Ethiopia
Source: PLoS One. 2025 Feb 7;20(2):e0318242. doi: 10.1371/journal.pone.0318242 (PMC12140114; doi:10.1371/journal.pone.0318242)
Supplement: S3 Fig — Legend: VRE, vancomycin resistant Enterococci; MIC: minimum inhibition concertation. (PDF) [file pone.0318242.s003.pdf]

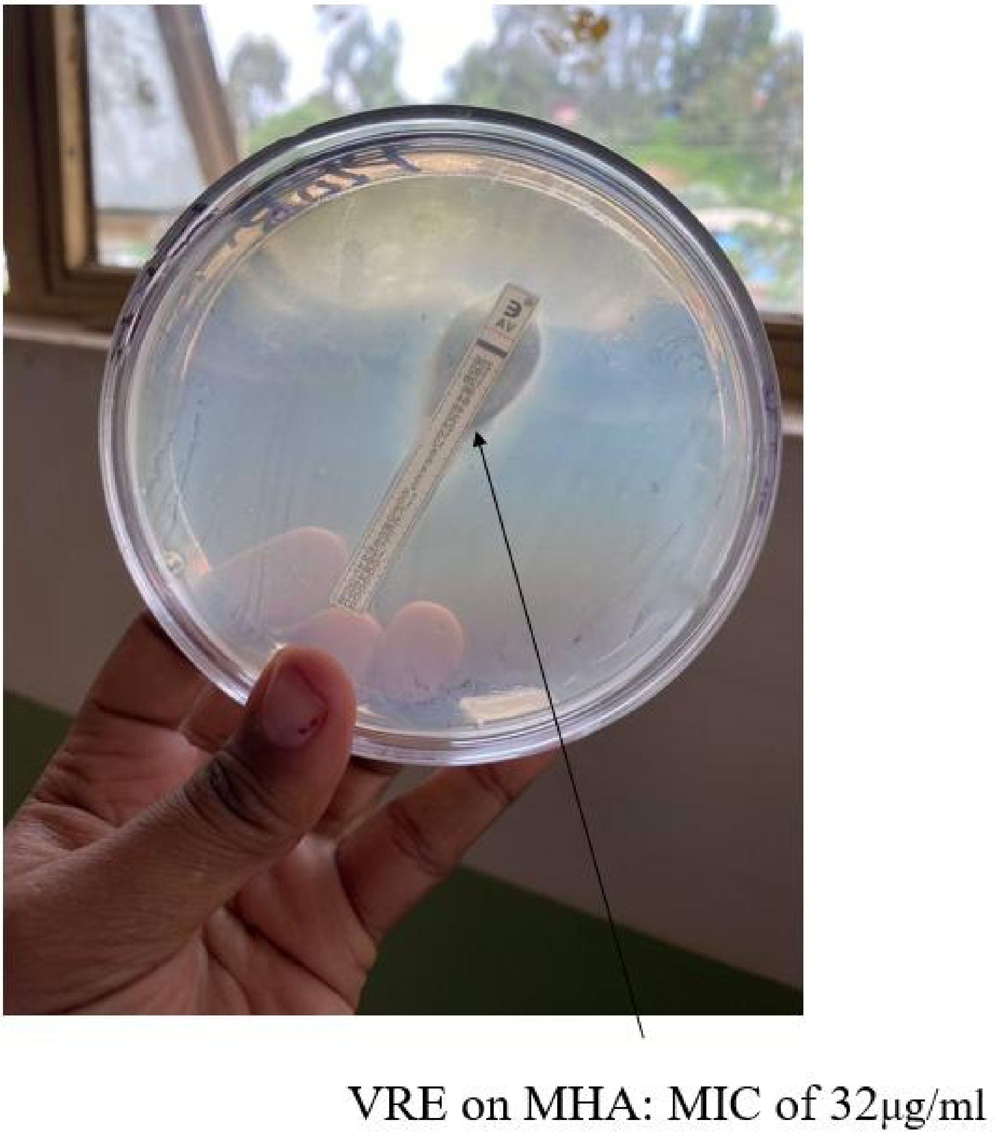

S3 Fig. VRE on Muller Hinton agar, MIC of 32μg/ml. Legend: VRE, vancomycin resistant *enterococci*; MIC: minimum inhibition concertation.
