# Supplementary material for: Colonization of methicillin-resistant Staphylococcus aureus and vancomycin-resistant Enterococci and its associated factors in cancer patients at the University of Gondar Comprehensive Specialized Hospital, Northwest Ethiopia
Source: PLoS One. 2025 Feb 7;20(2):e0318242. doi: 10.1371/journal.pone.0318242 (PMC12140114; doi:10.1371/journal.pone.0318242)
Supplement: Attachment — Submitted filename: plose one coment.pdf [file pone.0318242.s007.pdf]

Tittle: Methicillin-resistant Staphylococcus aureus and Vancomycin-resistant Enterococci species colonization among cancer patients at the University of Gondar Comprehensives Specialized Hospital, Northwest Ethiopia

Comments

The authors in this paper have taken an attempt to report the Methicillin-resistant Staphylococcus aureus and Vancomycin-resistant Enterococci species colonization among cancer patients at the University Of Gondar Comprehensives Specialized Hospital, Northwest Ethiopia This is of course is very interesting subject which creates interest among readers. However the paper seems very poor standard and not at par for international readers which must be revised before publication based on the following comments

Major comments:

1. I suggest the tittle should be corrected as follows "colonization rate of Methicillin-resistant Staphylococcus aureus and Vancomycin-resistant Enterococci species among cancer patients at the University of Gondar Comprehensives Specialized Hospital, Northwestern Ethiopia".
2. The authors should be remove all author information (email addresses) except cross ponding authors from the cover page.
3. **In abstract section:**

   -what was the biochemical test used to identify the *S. aureus* and Enterococci species? Please include this in method section.

   -Line 32: it says: Nasal swab and fecal specimens were collected and inoculated onto mannitol salt agar and bile esculin agar and incubated at 37°C and **43-45°C.** Is the incubate has range or fixed value, please use fixed numerical value either 43, 44 or 45 **°C** Instead of using range value.

   -line 34-37: It says: "To detect methicillin-resistant Staphylococcus aureus, a Cefoxitin disk was used. Inducible clindamycin resistance in Staphylococcus aureus was determined by the D test. Epi-info version 7 and Statistical Package for the Social Sciences version 27 were used for data entry and data analysis". Please correct as follows: To detect methicillin-resistant Staphylococcus aureus, a Cefoxitin disk was used. Inducible clindamycin resistance in Staphylococcus aureus was determined by the D test.

Epi-info version 7 and Statistical Package for the Social Sciences version 27 were used for data entry and data analysis, respectively."

-line 44-47: the authors should be including the adjusted odds ratio with p-value.

- The authors should be including the recommendation in abstract section.

4. **Introduction section**:

-the authors should include the magnitude of antimicrobial resistance of *S. aureus*, Enterococci species, and MRSA in Ethiopia especially in the study area.

5. **Materials and methods:**

- Line 108-111: it says: "The UoGCSH is one of the largest tertiary referral and teaching hospitals in the region, serving over 13 million residents in northwestern Ethiopia. Among other services, the established cancer treatment center provides services for cancer cases. The cancer treatment center has outpatient and inpatient departments with 40 beds each in pediatric and adult oncology". –the author should be provide the source of this information or reference is needed.

- The Source population and Study population seems to the same. The authors should be stated clearly.

- Sample size determination correction formula should be indicated to know your sample size is 288.

- Inclusion and exclusion criteria not clearly stated, is author only used the study participants newly diagnoses CA patients? Or included the CA patients in treatment stage?

-sampling technique should be convent sampling technique. If you are using stratified systematic random sampling, Please show us in figure how you are going to use it.

-why authors did not identify Enterococci species at species level?

- Why authors did not identify VRSA?

- In Antibiotic susceptibility test section: the authors should be mentioned the antimicrobial disk manufacturer company and country.

-line 205: put reference here

-For Methicillin-resistant *Staphylococcus aureus* identification: the authors should be include interpretation how to categorized MRSA OR MSSA

-The authors should be rewrite line 211-213.

-Ethical consideration: -please state the protocol number, date, month, and year.

-what was the fate of the positive individuals, are they treated or not?

6. **Results section:**

- Subheading should correct as follows "Socio-demographic characteristics of study participants" for Socio-demographic characteristics and "Clinical characteristics study participants" for Clinical characteristics.
- The presentation of MDR in Table 5 and Table 6 does not show the real MDR. So, the authors should be reanalysis MDR result as R1, R2, and R3…….or by listing the antimicrobials. Generally the authors do not analyze MDR isolates.
- Table 6 should be revised for example percentage is not indicated

7. **Discussion section:**

- The discussion should be improved with clear justification for example at end of discussion line 529- 533.

8. **Limitations of the study:**

-The authors should be put the limitations of the study before conclusion**.**

9. **Conclusion:**

-The conclusion is very shallow, so, please improve it.

10. The authors should be write recommendation. Research without recommendation is unusual.

11. There is old reference like 2008, so, please use the update one.

- Finally there are topographical, editorial, and grammatical errors. Please rewrite
