# Supplementary material for: Colonization of methicillin-resistant Staphylococcus aureus and vancomycin-resistant Enterococci and its associated factors in cancer patients at the University of Gondar Comprehensive Specialized Hospital, Northwest Ethiopia
Source: PLoS One. 2025 Feb 7;20(2):e0318242. doi: 10.1371/journal.pone.0318242 (PMC12140114; doi:10.1371/journal.pone.0318242)
Supplement: Attachment — Submitted filename: Response to Reviewers.docx [file pone.0318242.s008.docx]

Date: October, 2024

**To**: PLoS One

Editor-In-Chief

**From**: Authors

**Submission ID**: **PONE-D-24-24955**

**Title**: **Colonization of methicillin-resistant *Staphylococcus aureus* and vancomycin-resistant *Enterococci* and its associated factors in cancer patients at the University of Gondar Comprehensive Specialized Hospital, Northwest Ethiopia.**

**Authors` response to Editor**:

**Authors` response**: Dear Editor, we are grateful for the opportunity to revise our manuscript for PLoS One and appreciate the assignment of qualified reviewers. Their insightful comments significantly enhanced our work.

**Editorial comment**: Please ensure that your manuscript meets PLOS ONE's style requirements, including those for file naming.

**Authors` response:** Thank you for your feedback; we have made the necessary revisions.

**Editorial comment:** We suggest you thoroughly copyedit your manuscript for language usage, spelling, and grammar. If you do not know anyone who can help you do this, you may wish to consider employing a professional scientific editing service.

**Authors` response:** Thank you for your insightful comment. We have revisited the manuscript for copyediting and made several corrections, with significant assistance from two foreign language professors specializing in English.

**Editorial comment:** Please include captions for your Supporting Information files at the end of your manuscript, and update any in-text citations to match accordingly. Please see our Supporting Information guidelines for more information

**Authors` response:** Thank you for your feedback; we have closely followed your guidelines and made the necessary corrections.

**Authors` response to Reviewer #1**

**Reviewer #1 comment**: Firstly, I want to note that the discussion part has 7 pages and difficult to find the key message of the article. I think it should be rewritten having its key findings.

**Authors` response:** Although the discussion surrounding the results was quite extensive and detailed, it was crucial to engage in this thorough examination due to the numerous objectives that the study aimed to address. Our previous discussion lacked clarity on key points, but we have since improved our communication by incorporating your insightful comment for clearer, more understandable writing.

**Reviewer #1 comment**: Line 22-23 “However, the prevalence of these pathogens among cancer cases in Northwestern Ethiopia remains unreported.” I read some papers about methicillin resistance *S. aureus* in nasal swap in the study area. I think it needs revision like: However, the prevalence of………. under-reported.

**Authors` response:** Thank you for your feedback; we have accepted this and corrected it as follows: the prevalence of these pathogens among cancer cases in Northwestern Ethiopia remains underreported. We cited the earlier statement because, despite a 2020 report on MRSA colonization alone, no study has examined both vancomycin-resistant *Enterococcus* and methicillin-resistant *Staphylococcus aureus* colonization in this population in the area.

**Reviewer #1 comment**: Line 32: Are you sure that the incubation temperature of the stated organism is at 43-45°C?

**Authors` response:** *Enterococcus* can grow at temperatures ranging from 37°C to 45°C. We specifically selected 43-45°C for our study to selectively identify *Enterococci* in fecal specimens. This higher temperature inhibits the growth of other enteric species, creating a selective environment that favors *Enterococci*, as many organisms in fecal samples can thrive at 37°C.

**Reviewer #1 comment**: Line 54: “Cancer patients…..” already stated in line 53. I think you should replace it by “They..”

**Authors` response:** Thank you for your feedback; we have accepted this and corrected it as follows: They are at risk for infections and colonization due to various factors such as chemotherapy, neutropenia, indwelling central venous catheters, frequent antimicrobial administration, frequent hospitalization, and surgical interventions.

**Reviewer #1 comment**: Line 205:” ………resistant based on the CLSI 2022 guideline”. Please cite the reference

**Authors` response:** We have accepted the feedback and made the necessary corrections.

**Reviewer #1 comment**: Line 208 “……. incubated at 37 °C…” Are you sure that the incubation temperature for MRSA is 37°C? Please revise it.

**Authors` response:** We have accepted the feedback and made the necessary corrections. The incubation time for isolation of *S. aureus* on mannitol salt agar was 37°C but the incubation temperature of antimicrobial susceptibility pattern for MRSA was 35°C.

**Reviewer #1 comment**: Line 254: “….. 288 cancer study participants were included.” It requires rewriting. For example, ……Cancer patients were included or simply study participants…...

**Authors` response:** We have acknowledged and corrected the comment. In this study, 288 study participants were included.

**Reviewer #1 comment**: Line 381-383: “….Cancer treatments such as chemotherapy can weaken the body's ability to fight infections…..”. How do you know? How do you convince the reader? It is better to reference it to prove to the reader.

**Authors` response:** We have acknowledged and corrected the comment.

**Reviewer #1 comment**: Line 437: “…participants however,…” please put ; before however, or full-stop then begin with uppercase However….

**Authors` response:** We have accepted the feedback; however, since several reviewers instructed us to focus on key findings, we have omitted that finding from the discussion section.

**Reviewer #1 comment**: Line 439: by Debre Markos…. I think, it's better to say in Debre Markos

**Authors` response:** We have accepted the feedback; however, since several reviewers instructed us to focus on key findings, we have omitted that finding from the discussion section.

**Reviewer #1 comment**: Based on your findings, any key message was not forwarded to the readers and the community. I advise the authors to add the recommendation in the conclusion part.

**Authors` response:** We have accepted your feedback and incorporated your valuable suggestions.

**Authors` response to Reviewer #2**

**Reviewer #2 comment**: The current study is very interesting; but it needs a major revision and the authors should address the following comments to improve the quality of the manuscript:

**Authors` response:** Thank you for your clear and constructive feedback, which is invaluable in helping us improve our work. We want to assure you that we have made every effort to address the comments you provided.

**Reviewer #2 comment**: Please write the scientific names of bacterial pathogens in the correct form all over the manuscript and in the References section (should be italic).

**Authors` response:** We have accepted the feedback and made the necessary corrections.

**Reviewer #2 comment**: The manuscript should be revised for grammar mistakes.

**Authors` response:** We have accepted the feedback and made the necessary corrections.

**Reviewer #2 comment**: Please modify the title. Include associated factors on it

**Authors` response**: we have accepted the comment and modified the title accordingly.

Colonization of methicillin-resistant *Staphylococcus aureus* and vancomycin-resistant *Enterococci* and its associated factors in cancer patients at the University of Gondar Comprehensive Specialized Hospital, Northwest Ethiopia.

**Reviewer #2 comment**: is it enough mannitol salt agar and bile esculin agar to identify those species????????????????

**Authors` response:** We initially identified *S. aureus* using mannitol salt agar, which allowed us to determine its affinity for the *Staphylococcus* genus by growing in a high salt environment and indicating fermentation of mannitol. The species was confirmed by a positive coagulase test with sheep blood plasma.

For the identification of *Enterococcus* species, the specimen was initially inoculated onto bile esculin agar. Although bile esculin agar is not a fully selective medium and can support the growth of other Gram-negative bacteria, subsequent Gram staining tests confirmed the presence of *Enterococcus* species. This was further supported by the catalase test, which yielded a negative result. The combination of these tests—Gram reaction and catalase activity—provided a strong confirmation of the presence of *Enterococcus*, reinforcing the initial findings obtained from the bile esculin agar.

Due to the diverse biochemical characteristics of *Enterococcus* species and the lack of other biochemical reactions applicable to all the enterococci species, our study only utilized growth characteristics, Gram reaction, and catalase reaction tests.

**Reviewer #2 comment**: on the abstract part you must include 95 % CI for each point prevalence of your findings and also please include AOR with p-value for the associated factors, and add more details about the used methods and most prevalent results in the abstract.

**Authors` response:** We have acknowledged the comment and made the necessary revisions.

**Reviewer #2 comment**: Introduction: (This part needs more work and synthesis of ideas):

**Authors` response:** We have incorporated all the reviewer’s comment on the introduction section and tried to make it better accordingly

**Reviewer #2 comment**: added reference on line 90 and 91 about definitions of Multi-drug resistance (MDR).

**Authors` response:** We have acknowledged the comment and cited reference accordingly.

**Reviewer #2 comment**: Give a hint about the virulence factors and the mechanism of disease occurrence of pathogens.

**Authors` response:** We appreciate your insightful feedback, and in the introductory section, we have attempted to provide a concise overview of the virulence factors and the mechanism of disease occurrence of both MRSA and VRE.

**Reviewer #2 comment**: The authors should illustrate the public health importance concerning the magnitude, mortality, morbidity in details.

**Authors` response:** Thank you for your insightful comment; we have added information on the public health significance of MRSA and VRE mortality and morbidity in the introduction section.

**Reviewer #2 comment**: On line 104 delete A cross-sectional institution-based study and replaced by An institution-based cross-sectional study

**Authors` response:** We have acknowledged the comment and made the necessary revisions as An institution-based cross-sectional study was carried out at the University of Gondar Comprehensive Specialized Hospital (UoGCSH) oncology treatment center from May 1 to July 30, 2023

**Reviewer #2 comment**: On sample size determinations, are you sure is not recent study on your study title, rechecked it. And recalculate it ???????

**Authors` response:** In studies with two objectives, the choice of which objective's p-value to use for sample size calculations the objective associated with the higher p-value is often selected as the basis for sample size determination. This approach is grounded in the need to ensure adequate power for detecting clinically meaningful differences, as a higher p-value may indicate a less stringent criterion for significance, potentially requiring a larger sample size to achieve the desired power [1].

The current study had two objectives

1. Prevalence of MRSA colonization

For this particular objective, A 2020 study investigated MRSA colonization in cancer patients in the study area that reported an MRSA colonization prevalence of 6.5% [2]

2. Prevalence of VRE colonization

This objective lacks prior studies on the same population, so a p-value of 0.5 was selected to increase the required sample size, a common practice in the absence of previous research.

When a study has two objectives, the one with the higher p-value typically determines the sample size. Therefore, we selected the second objective with a prevalence of 0.5 for our calculations.

**Reviewer #2 comment**: How to stratified your study participants and include number of study participants in each starata

**Authors` response:** The UoGCSH oncology treatment center treats approximately 2,800 patients annually. This estimate is based on a review of patient flow in the three months preceding the data collection period of this study, during which an estimated 800 patients requested treatment in the UoGCSH oncology treatment center.

Stratified systematic random sampling was used to include study participants as

Pediatric oncology inpatient: Cancer patients aged ≤ 18 years admitted to the children's ward for chemotherapy; N.B:- The UoGCSH Pediatrics oncology ward does not have a proper OPD so the all the pediatrics study participants were included were inpatients.

Adult oncology inpatient: Cancer patients aged >18 years admitted to the adult oncology ward for chemotherapy

Adult oncology OPD: cancer patients aged >18 years receiving follow-up and routine examinations without undergoing chemotherapy

A systematic random sampling method was used to recruit study participants from the above-mentioned stratum.

Probability proportional to size /PPS (proportional allocation technique) was obtained by using the formula Nf= (ni /N*n) =Nf= Average no of each ward patients ×total sample size

Total admitted patients in all wards

Pediatrics Inpatient = 83 x 288= 29.8≈30

800

Adult Inpatient ward n =390 x 288=140

800

Adult Outpatient n=327 x 288 =117.7≈118

800

Systematic random sampling technique was used to include the study participants by calculating kth value, where N= 800 (based on the average data obtained from three month’s previous performance of the hospitals admission); adult OPD (140), adult inpatient (118), pediatric inpatient (30), Then based on the year of 2023 within three consecutive months (May to July, 2023) from UoGCSH oncology treatment center, the sample size was recruited based on this technique and the study participants was included in every Kth value.

Kth value of Adult OPD = 327/118= 2.7≈3

Kth value of Adult inpatient= 390/140= 2,78≈ 3

Kth value of Pediatrics inpatient=83/30≈ 2.7≈3

**Reviewer #2 comment**: Why you exclude patients who had an upper respiratory tract infection or who showed signs of coughing or difficulty breathing within the last three consecutive months, and patients who did not have updated whole blood cell reports were excluded from the study?

**Authors` response:** We excluded patients with upper respiratory infections or signs of coughing or breathing difficulty within the preceding three months, as they could inaccurately represent individuals who are solely colonized with MRSA; these patients might have been previously infected and now in a carrier state, whereas our study required participants to be exclusively colonized.

Whole blood cell reports are vital for cancer patients, as chemotherapy can weaken their immune systems. In our study, it was essential to evaluate their WBC status and its impact on potential colonization. Due to budget constraints, we utilized the routine updated whole blood count report which was ordered by their physicians to monitor participant progress.

**Reviewer #2 comment**: Rewrite Specimen collection and transportation in the correct way.

**Authors` response:** We value your insightful feedback, and we have updated the writing to reflect your suggestions.

**Reviewer #2 comment**: Specific references should be added to all the used methods and techniques.

**Authors` response:** We value your insightful feedback, and we have updated the reference accordingly.

**Reviewer #2 comment**: Discuss in detail the methods of isolation and identification of bacterial pathogens. Besides, specific references should be added.

**Authors` response:** We value your insightful feedback. A more detailed discussion of the isolation and identification of bacterial pathogens is now included in the methods section.

**Reviewer #2 comment**: Add the company, city, and country of the used bacterial media and reagents that were used in the biochemical identification of isolates.

**Authors` response**: We have accepted the comment and incorporated the company, city, and country of the used bacterial media and reagents that were used in the biochemical identification of isolates.

**Reviewer #2 comment**: Antimicrobial susceptibility testing, add the names of the antimicrobial classes of the tested antibiotics.

**Authors` response:** We value your insightful feedback and we have added the names of the antimicrobial classes of the tested antibiotics in the writing.

**Reviewer #2 comment**: PCR-based detection of antibiotic resistance genes in the recovered isolates should be carried out.

**Authors` response:** In our study, we agree that PCR-based detection of antibiotic resistance genes would have made the findings more reliable and accurate. However, due to budgetary limitations, we were only able to conduct phenotypic antimicrobial susceptibility testing. This limitation is acknowledged in the limitations section of our manuscript. We have also recommended that future studies in this area utilize the more robust PCR-based methodologies to assess antibiotic resistance genes directly.

**Reviewer #2 comment**: Results: rewrite again focus on major finding

**Authors` response:** Thank you for your feedback. We previously highlighted significant findings essential for readers, and in response to feedback, we have tried to summarize our results without leaving out essential information.

**Reviewer #2 comment**: Discuss in detail the phenotypic characters of the isolates.

**Authors` response:** We previously conducted a detailed analysis of the isolates' phenotypic characteristics, including growth traits, morphological features, and antibiotic susceptibility patterns. In response to your constructive comment, we have acknowledged the need to strengthen our justification for these susceptibility patterns and have made efforts to do so.

**Reviewer #2 comment**: The detection of antimicrobial resistance genes in the retrieved isolates should be carried out if applicable.

We appreciate your valuable insights. Although budgetary constraints limited our ability to detect antimicrobial resistance genes, we believe that routine phenotypic antimicrobial resistance data provided significant insights. By transparently reporting this limitation and suggesting improvements for future research, we aimed to contextualize our study’s findings. The isolates have been properly stored for future use, and we will pursue the detection of antimicrobial genes when a suitable opportunity arises.

**Reviewer #2 comment**: The correlation between the phenotypic and genotypic MDR should be performed.

**Authors` response:** Thank you for your feedback. We recognize that incorporating advanced genomic techniques could significantly enhance investigations by deepening our understanding of the connections between phenotypic expressions and genetic mechanisms. However, due to budget constraints, we were unable to conduct genotypic tests. However we do recommend that this be addressed in future studies.

**Reviewer #2 comment**: Discussion; the authors are advised to illustrate the real impact of their findings without repetition of results.

**Authors` response:** We apologize for not clearly communicating the impact of our study previously. Our previous discussion lacked clarity on key points, but we have since improved our communication by incorporating your insightful comment for clearer, more understandable writing. Thank you for highlighting this for us to improve.

**Reviewer #2 comment**: Illustrate the different mechanisms of antimicrobial resistance in Methicillin-resistant *Staphylococcus aureus* and Vancomycin-resistant *Enterococci* species

**Authors` response:** We aimed to provide a brief introduction to the mechanisms of antimicrobial resistance in Methicillin-resistant *Staphylococcus aureus* and Vancomycin-resistant *Enterococci*, specifically in paragraph 3 of the introduction section.

**Reviewer #2 comment**: Conclusion should be rephrased to be sounded. A real conclusion should focus on the question or claim you articulated in your study.

**Authors` response:** We have noted the comment and made the necessary revision.

**Authors` response to Reviewer #3**

**Reviewer #3 comment**: The authors in this paper have taken an attempt to report the Methicillin-resistant *Staphylococcus aureus* and Vancomycin-resistant *Enterococci* species colonization among cancer patients at the University of Gondar Comprehensive Specialized Hospital, Northwest Ethiopia. This is, of course, a very interesting subject that creates interest among readers. However, the paper seems to be of very poor standard and not at par for international readers, which must be revised before publication based on the following comments.

**Authors` response:** Thank you for your insightful comment; we have implemented your suggestion, along with those of the other reviewers, to enhance its suitability for publication.

**Reviewer #3 comment**: I suggest the tittle should be corrected as follows “colonization rate of Methicillin resistant *Staphylococcus aureus* and Vancomycin-resistant *Enterococci* species among cancer patients at the University of Gondar Comprehensives Specialized Hospital, Northwestern Ethiopia”.

**Authors` response:** We acknowledge you to remind this point to our focus. However, when we started this study we did a thorough research on what the difference between prevalence of colonization and the prevalence of colonization rate means and by following previous studies that were done and we came to conclude the difference between the two.

**Prevalence of colonization**: It refers to the proportion or percentage of a population that is colonized by a particular organism at a given point in time. It represents the overall burden or extent of colonization within the population [3].

**Prevalence of colonization rate**: The prevalence of colonization rate refers to the frequency of colonization events within a population over time, indicating how quickly new colonization instances occur, often quantified as the number of new colonization per unit of time [4].

Our study is focused on a certain period of time, so we felt it was fair to refer to colonization or prevalence of colonization rather than "colonization rate". We have provided citations for studies that conducted their research using definitions that are comparable to ours.

**Reviewer #3 comment**: The authors should be remove all author information (email addresses) except corresponding authors from the cover page.

**Authors` response:** We have accepted the comment and updated the cover page accordingly.

**Reviewer #3 comment**: In abstract section, what was the biochemical test used to identify the *S. aureus* and *Enterococci* species? Please include this in method section.

**Authors` response:** We have acknowledged the comment and made the necessary revisions.

**Reviewer #3 comment**: In abstract section, Line 32: it says: Nasal swab and fecal specimens were collected and inoculated onto mannitol salt agar and bile esculin agar and incubated at 37°C and 43-45°C. Is the incubate has range or fixed value, please use fixed numerical value either 43, 44 or 45 °C instead of using range value.

**Authors` response:** Yes, this comment is valid; specific temperatures are crucial for microorganism growth. However, one of our lab incubators was set to 43°C but frequently fluctuated, reaching up to 45°C. That's why we reported it as 43-45°C.

**Reviewer #3 comment**: line 34-37: It says: “To detect methicillin-resistant *Staphylococcus aureus*, a Cefoxitin disk was used. Inducible clindamycin resistance in *Staphylococcus aureus* was determined by the D test. Epi-info version 7 and Statistical Package for the Social Sciences version 27 were used for data entry and data analysis”. Please correct as follows: To detect methicillin-resistant *Staphylococcus aureus*, a cefoxitin disk was used. Inducible clindamycin resistance in *Staphylococcus aureus* was determined by the D test. Epi-info version 7 and Statistical Package for the Social Sciences version 27 were used for data entry and data analysis, respectively.”

**Authors` response**: We have acknowledged the comment and made the necessary revisions as “To detect methicillin-resistant *Staphylococcus aureus*, a cefoxitin disk was used. Inducible clindamycin resistance in *Staphylococcus aureus* was determined by the D test. Epi-info version 7 and Statistical Package for the Social Sciences version 27 were used for data entry and data analysis, respectively.”

**Reviewer #3 comment**: line 44-47: the authors should be including the adjusted odds ratio with p-value.

**Authors` response**: We have acknowledged the comment and made the necessary revisions.

**Reviewer #3 comment**: The authors should be including the recommendation in abstract section.

**Authors` response:** We have acknowledged the comment and made the necessary revisions.

**Reviewer #3 comment**: The authors should include the magnitude of antimicrobial resistance *of S. aureus*, *Enterococci* species, and MRSA in Ethiopia especially in the study area.

**Authors` response:** We respect your insightful comments and we have included a brief insight on the extent of antibiotic resistance of *S. aureus, Enterococci* species, and MRSA in Ethiopia notably in the study area.

**Reviewer #3 comment**: Line 108-111: it says: “The UoGCSH is one of the largest tertiary referral and teaching hospitals in the region, serving over 13 million residents in northwestern Ethiopia. Among other services, the established cancer treatment center provides services for cancer cases. The cancer treatment center has outpatient and inpatient departments with 40 beds each in pediatric and adult oncology”. –the author should be provide the source of this information or reference is

needed.

**Authors` response:** We have cited a reference and provided evidence showing that patient volume at UoGCSH is 13 million.

**Reviewer #3 comment**: The Source population and Study population seems to the same. The authors should be stated clearly.

**Authors` response:** Thank you for your feedback. We have updated the definition to clarify the difference.

**Reviewer #3 comment**: Sample size determination correction formula should be indicated to know your sample size is 288.

**Authors` response:** The sample size was determined using a single population proportion formula with 5% expected margins of error, 95% confidence interval (Za/2 = 1.96) and 50% prevalence because there are no similar study reports neither in the study area nor in the country Ethiopia on the prevalence of colonization of MRSA and VRE among cancer patients. Therefore, the sample size was determined as follows: N=sample size, P=0.5, Confidence interval=95%, d=5%

N=z2*p(1-p)/d2 = N=(1.96)2(0.5) *(1-0.5)/ (0.05)2 = N=385

Data records at the UoGCSH showed that a total of 800 patients visited the hospital as oncology case within the last 3 months. We guessed that the study would take 3 months by considering 22 working days per a month. Therefore, 800 patients were expected to visit the UoGCSH oncology treatment center during the study period. Since the expected number of patients being treated at the UoGCSH oncology center is less than 10,000, correction was required to adjust the final sample size and was determined as follows: nC= Corrected sample size nP= previously uncorrected sample size N= the number of patients expected during the study period

N=800

nP = 385

nC =	n	nC= 385	385	= 260.1	nC = 260.1≈261

1+ n	1 + 385	=	1.48

N	800

Considering 10% non-response rate the total sample size will be

261+26.1=287.1≈ 288

**Reviewer #3 comment**: Inclusion and exclusion criteria not clearly stated, is author only used the study participants newly diagnoses CA patients? Or included the CA patients in treatment stage?

**Authors` response:** We have aimed to include all cancer patients present during the study period, regardless of whether they are newly diagnosed or in the treatment phase. Our intention behind this approach was to investigate how factors such as the round of chemotherapy they are receiving, the frequency of their hospital visits, and the specific stage of their cancer may influence the outcomes of interest. Which varies in the type of cancer each study participants had, whether they are newly diagnosed or are in remission. By incorporating this broader spectrum of patients, we had hoped to gain a comprehensive understanding of how these variables interact and potentially impact the health of patients with weakened immune systems.

**Reviewer #3 comment**: sampling technique should be convent sampling technique. If you are using stratified systematic random sampling, Please show us in figure how you are going to use it.

**Authors` response:** We chose stratified systematic random sampling because the study participants were organized into strata: adult outpatient, adult inpatient, and pediatric inpatient. This method ensured fair representation from each group, considering their different participant flows, and reduced bias compared to random selection. Stratification allowed for effective analysis of variations in outcomes across these groups, which was essential for understanding the unique dynamics in adult and pediatric care. Overall, it enhanced the validity of our findings and ensured equitable treatment of all participants.

**UoGCMHS oncology treatment center (800)N for Three months**

Pediatric oncology (83)

Adult oncology (717)

Adult OPD (327)

Pediatrics Inpatient(83)

Adult Inpatient (390)

Proportional Sampling

n =118

n = 30

n = 140

Systematic random sampling

Figure 1: Sampling procedure: The diagrammatic presentation of the sampling procedure of patients at UoGCSH oncology treatment center, Northwest Ethiopia

**Reviewer #3 comment**: why authors did not identify *Enterococci* species at species level?

**Authors` response:** We accepted the feedback, but classifying *Enterococcus* at the species level requires molecular techniques, which we couldn't afford. While other controversial methods, such as biochemical tests and antimicrobial resistance, exist for identifying *Enterococcus faecalis* or *Enterococcus faecium*, we couldn’t confirm that the isolated *Enterococcus* was exclusively one of these two species. Other Enterococcus species have been documented in the country, albeit less frequently. Classifying *Enterococcus* solely as either *Enterococcus faecalis* or *Enterococcus faecium* could lead to misclassification of other species. Therefore, we opted to classify it at the genus level and store the specimen, hoping for accurate species differentiation in the future. We followed this approach in our study.

**Reviewer #3 comment**: Why authors did not identify VRSA?

**Authors` response:** We were grateful that no VRSA was recovered from this highly vulnerable population, as its presence would lead to more complex health issues and an increased risk of these strains circulating in the study area. Our hypothesis posited that gene transfer of vanA/vanB from vancomycin-resistant *Enterococcus* occurs when *S. aureus* and vancomycin-resistant *Enterococcus* share an ecological niche. Since no study participants were colonized by both *S. aureus* and vancomycin-resistant *Enterococcus*, either at the same or different sites (such as nasal and gastrointestinal), we suspect this may explain our findings.

**Reviewer #3 comment**: In Antibiotic susceptibility test section: the authors should be mentioned the antimicrobial disk manufacturer company and country.

**Authors` response:** We acknowledge the feedback and we have made the necessary revision.

**Reviewer #3 comment**: line 205: put reference here

**Authors` response:** We have accepted the comment have cited a reference.

**Reviewer #3 comment**: For Methicillin-resistant *Staphylococcus aureus* identification: the authors should be include interpretation how to categorized MRSA OR MSSA

**Authors` response:** We have taken the comments under consideration and made the required revisions.

**Reviewer #3 comment**: The authors should be rewrite line 211-213.

**Authors` response:** We have taken the comments under consideration and made the required revisions.

**Reviewer #3 comment**: Ethical consideration: -please state the protocol number, date, month, and year.

**Authors` response**: We have taken the comments under consideration and made the required revisions.

**Reviewer #3 comment**: what was the fate of the positive individuals, are they treated or not?

**Authors` response:** Given the complex medication regimens cancer patients often face and the risks of VRE decolonization in the gut, we provided the physicians with the necessary results by the end of the study for better management of patients. This enabled them to customize antibiotic treatments, according to the antimicrobial susceptibility pattern of the isolated organisms that benefited the patients without exacerbating side effects from other chemotherapy drugs.

**Reviewer #3 comment**: Subheading should correct as follows “Socio-demographic characteristics of study participants” for Socio-demographic characteristics and “Clinical characteristics study participants” for Clinical characteristics.

**Authors` response**: We have taken the comments under consideration and made the required revisions.

**Reviewer #3 comment**: The presentation of MDR in Table 5 and Table 6 does not show the real MDR. So, the authors should be reanalysis MDR result as R1, R2, and R3…….or by listing the antimicrobial. Generally the authors do not analyze MDR isolates.

**Authors` response:** We had previously analyzed the MDR results as discussed but omitted them from the manuscript to prevent overcrowding of tables. We have now revisited and updated the original MDR table.

**Reviewer #3 comment**: Table 6 should be revised for example percentage is not indicated.

**Authors` response:** We have taken the comments under consideration and revised it accordingly.

**Reviewer #3 comment**: The discussion should be improved with clear justification for example at end of discussion line 529- 533.

**Authors` response:** Thank you for your valuable suggestions; they have been instrumental in refining our work. We have considered your comments and improved the discussion section to include a clear key message and expand on the implications of our findings to provide a broader context for our work at the last paragraph of the discussion section.

**Reviewer #3 comment**: The authors should be put the limitations of the study before conclusion.

**Authors` response:** We acknowledge the comment and have made the necessary revision.

**Reviewer #3 comment**: The conclusion is very shallow, so, please improve it.

**Authors` response:** Thank you for your insightful remarks. We have revised the conclusion to offer a more comprehensive analysis of the findings.

**Reviewer #3 comment**: The authors should be write recommendation. Research without recommendation is unusual.

**Authors` response:** Thank you for your insightful feedback we have constructed a recommendation based on the findings of our study.

**Reviewer #3 comment**: There is old reference like 2008, so, please use the update one.

**Authors` response:** We have updated our previous reference, but we had to use two distinct sources: one for the normal WBC range for patients aged 1 year to 5 years from a 2009 study, and another for the normal ANC range for patients aged 1 to 5 years from a 2010 study. This was necessary because there are no published studies on white blood cell count and absolute neutrophil count ranges for children aged 1 to 5 in our country or neighboring countries. Additionally, we did not use the standard range as it would not be appropriate given our country's economic conditions and living habits. We acknowledge that citing two different sources and studies over a decade old may raise questions about consistency; however, our priority is to ensure our references reflect the most relevant data for our region's unique demographic and health profile. Given the significant variability in immune function and health status among children, particularly those from diverse socioeconomic backgrounds, we felt it imperative to adapt this reference ranges however old to better suit our context rather than using a standard range which is more suitable for European standard.

**Reviewer #3 comment**: Finally, there are topographical, editorial, and grammatical errors. Please rewrite

**Authors` response:** Thank you for your insightful feedback. We have updated the entire manuscript making sure there are no topographical, editorial and grammatical errors as much as possible.

**Reference**

1.	Wang H, Chow SC. Sample size for comparing proportions*.* Methods and Applications of Statistics in Clinical Trials: Planning, Analysis, and Inferential Methods 2014;2:653-63.

2.	Tigabu A, Belay G. Inducible Clindamycin and Methicillin Resistant *Staphylococcus aureus* Among Cancer Patients at University of Gondar Compressive Specialized Hospital, Northwest Ethiopia: Carriage Rate and Antibiotic Resistance Patterns*.* *Clinical Laboratory* 2020.

3.	Armin S, Azimi L, Shariatpanahi G, Shirvani A, Tehrani NA. The Prevalence of Colonization with Carbapenem-resistant *Enterobacteriaceae*, Escherichia coli, Klebsiella and Enterobacter, and Related Risk Factors in Children*.* *Archives of Pediatric Infectious Diseases* 2023;11.

4.	Pitiriga V, Bakalis J, Theodoridou K, Dimitroulia E, Saroglou G, Tsakris A. Comparison of microbial colonization rates between central venous catheters and peripherally inserted central catheters. *Antimicrobial Resistance & Infection Control* 2023;12:74.
