# Supplementary material for: Colonization of methicillin-resistant Staphylococcus aureus and vancomycin-resistant Enterococci and its associated factors in cancer patients at the University of Gondar Comprehensive Specialized Hospital, Northwest Ethiopia
Source: PLoS One. 2025 Feb 7;20(2):e0318242. doi: 10.1371/journal.pone.0318242 (PMC12140114; doi:10.1371/journal.pone.0318242)
Supplement: Attachment — Submitted filename: Response_to_Reviewers_auresp_2.docx [file pone.0318242.s009.docx]

Date: December, 2024

**To**: PLoS One

Editor-In-Chief

**From**: Authors

**Submission ID**: **PONE-D-24-24955**

**Title**: **Colonization of methicillin-resistant *Staphylococcus aureus* and vancomycin-resistant**

***Enterococci* and its associated factors in cancer patients at the University of Gondar**

**Comprehensive Specialized Hospital, Northwest Ethiopia.**

**Reviewer #2 comment**: This manuscript is markedly improved from the last time I reviewed it. I thank the authors for their efforts and especially for the improved. I continue to have some concerns about the data that should be addressed in the methods and limitations sections of the manuscript.

**Authors` response:** Thank you. Your insightful comment significantly improved the manuscript's quality.

**Reviewer #2 comment**: On abstract: please include the gaps rather than prevalence also include others.

**Authors` response:** We have tried to use the given amount of word limit for the abstract section to wisely summarize our study but adding more information will exceed the already set out word limit .

**Reviewer #2 comment**: L---21 delete at the University of Gondar Comprehensive Specialized Hospital b/s you stated it on the objective part, however include your sample size, sampling technique, and populations.

**Authors` response:** We accepted the feedback and revised the text to: A cross-sectional study enrolled 288 confirmed cancer participants through stratified systematic random sampling, gathering socio-demographic and clinical data via pretested structured questionnaires from May 1 to July 30, 2023.

**Reviewer #2 comment**: L---22 add pretest on structured questionnaires

**Authors` response:** We accepted the comment and revised it accordingly.

**Reviewer #2 comment**: L—23 include method and way of transportations of your sample collections.

**Authors` response:** We appreciate the feedback and have included the methods and transportation details for specimen collection. Each participant provided two specimens: a nasal swab and a fecal sample. Specimen containers were labeled with unique identifiers, including specimen number, date, type, and time of collection. Nasal swabs were collected using sterile swabs, inserted at least 1 cm into each nostril, and rotated against the nasal membrane for 10 to 15 seconds. Both nostrils were sampled with the same swab, which was then placed in Amies transport medium. Fecal specimens were collected in leak-proof plastic containers, swabbed, and transferred to Cary Blair transport medium**.**

**Reviewer #2 comment**: Can directly inoculate the specimens in to manitol salt agar and Bile esculin agar after you collect the sample.

**Authors` response:** We identified *S. aureus* and *Enterococci* by directly inoculating specimens from the transport medium onto Mannitol salt agar and Bile esculin agar, respectively. This direct inoculation, along with sub culturing, biochemical tests, and Gram staining, ensured accurate identification of these organisms.

**Reviewer #2 comment**: Pearson Chi-Square, bivariable and multivariable logistic regression analyses identified associations, with a p-value < 0.05 considered statistically significant. Rewrite again it

**Authors` response:** We accepted the feedback and revised the text to: Pearson Chi-Square was initially used to assess the association between factors and outcomes, with a p-value cutoff of < 0.05 for statistical significance. This was followed by bivariable and multivariable logistic regression analyses to evaluate the strength of those associations, using p-value cutoffs of < 0.2 in the bivariable analyses and p value of < 0.05 cutoff for significance in multivariable analyses, respectively.

**Reviewer #2 comment**: When you use *S. aureus* first write the full name, then you must use the abbreviations all over the documents and the same true for *enterococcus* to its consistence.

**Authors` response:** We have implemented the feedback and improved *S. aureus*. However, we decided to use "*enterococci*" throughout the manuscript, as it refers to the plural form, while "*enterococcus*" denotes the singular species.

**Reviewer #2 comment:** Introductions: it seem correct.

**Authors` response:** Your previous feedback, along with that of the other examiners, greatly improved the introduction.

**Reviewer #2 comment**: Methods and materials, in study setting include more about flow of cancer patients.

**Authors` response:** We accepted the feedback and made the necessary improvements.

**Reviewer #2 comment**: L----135 remove study period’s b/c you already mission on study setting.

**Authors` response:** We accepted the feedback and made the necessary improvements**.**

**Reviewer #2 comment**: It’s better to put the figure rather than word to explain your sampling procedure to indicate proportional allocations and indicates the number of participants from each strata.

**Authors` response:** We accepted the feedback and made the necessary improvements.

**Reviewer #2 comment**: What is the difference b/n your study populations and inclusions criteria???? Is it similar??????

**Authors` response:** Thank you for bringing this to our attention. We realize we haven't clearly defined these two terms. The study population includes patients referred to the UoGCSH oncology treatment center, who may have a possibility of cancer based on suggestive indications that need confirmation. In contrast, the inclusion criteria consist of patients with confirmed cancer cases.

**Reviewer #2 comment**: L--- 171-172 they have redundancy idea please rewrite it?

**Authors` response:** We accepted the feedback and made the necessary improvements.

**Reviewer #2 comment**: L –173- 176 it is better to move it in to data quality control part

**Authors` response:** Thank you for your valuable feedback; we have made the necessary improvements as advised.

**Reviewer #2 comment**: Generally you have to use pretested structured questioners to collect the data so, where you have gotten the questionnaire, and how to develop it and give different reference for it????

**Authors` response:** We took the original questionnaire developed by Mengesha et al. and made several modifications to ensure that it was appropriately tailored to fit the specific context and population that this particular study aims to examine [1].

**Reviewer #2 comment**: L--- 273-277 it needs modifications, please see in details.

**Authors` response:** We acknowledged the comment and made the required revisions.

Result and dissections

**Reviewer #2 comment**: It written in appropriate way but Write major finding for your result since it is very wide? And also discussions part.

**Authors` response:** We have shortened the results and discussion section in response to the feedback from all the examiners last time. While we have made further reductions, any more cuts would risk omitting essential information.

**Reviewer #2 comment**: I would like some discussion of their findings against past developments.

**Authors` response:** If the comment indicates a discussion of related studies, we have addressed this with multiple research, particularly the study by Tigabu et al., which focused on the same area and population [2].

**Reviewer #2 comment**: You are very much aware of the limitations of the study.

**Authors` response:** We have tried to clarify our study limitation transparently.

**Reviewer #2 comment**: Though it discovered in general nothing not known for the local situation it will be a good starting point to act.

**Authors` response:** Thank you for your valuable feedback. This study not only updates the prevalence of MRSA colonization but also presents the first findings on VRE colonization in the population. It provides important insights into the vancomycin susceptibility patterns of *S. aureus* and *Enterococci*, which have been previously overlooked due to a focus on Minimum Inhibitory Concentration (MIC) testing. Although VRSA was not detected, the high prevalence of VISA highlights the urgent need for physicians and future research to address this antibiotic resistance, especially since vancomycin is currently regarded as a last resort for treating gram-positive bacteria in this region and throughout Ethiopia.

**Reviewer #2 comment**: I suggest the authors to also add some references of data about other regions where those bacterial infections are endemic: Middle Est and Asia (India and South East Asia). A comparison between Ethiopia and those regions can make the study more interesting and relevant.

**Authors` response:** Thank you for your valuable feedback. The study examined the colonization of MRSA and VRE. We mainly compared the prevalence of these colonization with findings from similar global studies, noting that colonization and infection are different concepts and shouldn't be directly compared. While in analyzing antibiotic susceptibility patterns for *Enterococci* and *S. aureus*, we included studies that looked at both colonization and infection specifically those where bacterial infection are endemic, focusing mainly on understanding resistance patterns. We previously reduced the reference from past studies to not overcrowd and also picked out latest references.

**Reviewer #2 comment**: Finally, there are topographical, editorial, and grammatical errors. Please rewrite

**Authors` response:** We acknowledged the comment and made the required revisions.

**Reviewer #2 comment**: The conclusion is very shallow, so, please improve it.

**Authors` response:** We acknowledged the comment and made the required revisions.

**References**

1.	Mengesha TH, Ali MM, Mengistu M, Assegu Fenta D. High Gastrointestinal Colonization Rate of Vancomycin‐Resistant *Enterococci* among Hospitalized Patients: Potential Source for Resistant Gene*.* *International Journal of Microbiology* 2024;2024:6430026.

2.	Tigabu A, Belay G. Inducible Clindamycin and Methicillin Resistant *Staphylococcus aureus* Among Cancer Patients at University of Gondar Compressive Specialized Hospital, Northwest Ethiopia: Carriage Rate and Antibiotic Resistance Patterns*.* Clinical Laboratory 2020.
