# Supplementary material for: Colonization of methicillin-resistant Staphylococcus aureus and vancomycin-resistant Enterococci and its associated factors in cancer patients at the University of Gondar Comprehensive Specialized Hospital, Northwest Ethiopia
Source: PLoS One. 2025 Feb 7;20(2):e0318242. doi: 10.1371/journal.pone.0318242 (PMC12140114; doi:10.1371/journal.pone.0318242)
Supplement: Attachment — Submitted filename: Reviewer comment.docx [file pone.0318242.s005.docx]

**Reviewer Report**

**Comments**

Firstly, I want to note that the discussion part has 7 pages and difficult to find the key message of the article. I think it should be rewritten having its key findings.

**Abstract**

- Line 22-23 “However, the prevalence of these pathogens among cancer cases in Northwestern Ethiopia remains unreported.” I read some papers about methicillin resistance S.aureus in nasal swap in the study area. I think it needs revision like: However, the prevalence of………. under-reported.
- Line 32: Are you sure that the incubation temperature of the stated organism is at 43-45°C?

**Introduction**

- Line 54: “Cancer patients…..” already stated in line 53. I think you should replace it by “They..”

**Methodology:**

- Line 205:” ………resistant based on the CLSI 2022 guideline”. Please cite the reference
- Line 208 “……. incubated at 37 °C…” Are you sure that the incubation temperature for MRSA is 37°C? Please revise it.

**Results:**

- Line 254: “….. 288 cancer study participants were included.” It requires rewriting. For example, ……Cancer patients were included or simply study participants…...

**Discussion:**

The discussion part has 7 pages and difficult to find the key message of the article. I think it should be rewritten having its key findings.

- Line 381-383: “….Cancer treatments such as chemotherapy can weaken the body's ability to fight infections…..”. How do you know? How do you convince the reader? It is better to reference it to prove to the reader.
- Line 412: “…, However,….” please write in lowercase or replace the comma by full stop before it.
- Line 437: “…participants however,…” please put ; before however, or full-stop then begin with uppercase However….
- Line 439: by Debre Markos…. I think, it's better to say in Debre Markos

Conclusion:

- Based on your findings, any key message was not forwarded to the readers and the community. I advise the authors to add the recommendation in the conclusion part.
