# Supplementary material for: Colonization of methicillin-resistant Staphylococcus aureus and vancomycin-resistant Enterococci and its associated factors in cancer patients at the University of Gondar Comprehensive Specialized Hospital, Northwest Ethiopia
Source: PLoS One. 2025 Feb 7;20(2):e0318242. doi: 10.1371/journal.pone.0318242 (PMC12140114; doi:10.1371/journal.pone.0318242)
Supplement: Attachment — Submitted filename: Reviewer Comments.docx [file pone.0318242.s006.docx]

**Reviewer Comments**

| **It needs major revissions** | |
| --- | --- |
| The current study is very interesting; but it needs a major revision and the authors should address the following comments to improve the quality of the manuscript:  -Please write the scientific names of bacterial pathogens in the correct form all over themanuscript and in the References section (should be italic).  -The manuscript should be revised for grammar mistakes.  -Please modify the title. Include associated factors on it  - is it enough mannitol salt agar and bile esculin agar to identify those spcies????????????????  - on the abstract part you must include 95 % CI for each point prevalence of your findings and also please include AOR with p-value for the associated factors, and add more details about the used methods and most prevalent results in the abstract.  -Introduction: (This part needs more work and synthesis of ideas):  -added reference on line 90 and 91 about definations of Multi-drug resistance (MDR).  -Give a hint about the virulence factors and the mechanism of disease occurrence of pathogens.  - The authors should illustrate the public health importance concerning the magnitude, mortality, morbidity in drtails.  Material and methods:   - On line 104 delete A cross-sectional institution-based study and replaced by An institution-based cross-sectional study - On sample size determinations, are you sure is not recent study on your study title, rechecked it. And recalculate it ??????? - How to stratified your study participants and include number of study participants in each starata - Why you exclude patients who had an upper respiratory tract infection or who showed signs of coughing or difficulty breathing within the last three consecutive months, and patients who did not have updated whole blood cell reports were excluded from the study. - Rewrite Specimen collection and transportation In the correct way - Specific references should be added to all the used methods and techniques. - Discuss in detail the methods of isolation and identification of bacterial pathogens. Besides, specific references should be added. - Specific references should be added to all the used methods and techniques. - Add the company, city, and country of the used bacterial media and reagents that were used in the biochemical identification of isolates. - Antimicrobial susceptibility testing: - Add the names of the antimicrobial classes of the tested antibiotics. - PCR-based detection of antibiotic resistance genes in the recovered isolates should be carried out. - Results: rewrite again focus on major finding - Discuss in detail the phenotypic characters of the isolates. - The detection of antimicrobial resistance genes in the retrieved isolates should be carried out if applicable. - The correlation between the phenotypic and genotypic MDR should be performed, - Discussion: - The authors are advised to illustrate the real impact of their findings without repetition of results. - Illustrate the different mechanisms of antimicrobial resistance in Methicillin-resistant Staphylococcus aureus and Vancomycin-resistant Enterococci species   -Conclusion Should be rephrased to be sounded. A real conclusion should focus on the question or claim you articulated in your study. |  |
|  | |

Thank you All!!
